# Structural Behavior and Failure Characteristics of Fiber-Reinforced Polymer-Concrete Composite Beams Incorporating Glass Roving Tied GFRP Shear Connectors

**DOI:** 10.3390/polym17233201

**Published:** 2025-11-30

**Authors:** Ankit Singh Mehra, Shamsher Bahadur Singh, Venkatesh Kodur

**Affiliations:** 1 Department of Civil Engineering, Birla Institute of Technology and Science, Pilani, Pilani Campus, Vidya Vihar, Pilani 333031, Rajasthan, India; p20190440@pilani.bits-pilani.ac.in (A.S.M.); sbsingh@pilani.bits-pilani.ac.in (S.B.S.); 2 Department of Civil Engineering, Graphic Era Hill University, Dehradun 248002, Uttarakhand, India; 3 Centre for Promotion of Research, Graphic Era (Deemed to be) University, Dehradun 248002, Uttarakhand, India; 4 Department of Civil and Environmental Engineering, Michigan State University, 3546 Engineering Building, East Lansing, MI 48823, USA

**Keywords:** GFRP-concrete composite beam, GFRP shear connectors, pultruded GFRP profile, four-point bending test, interfacial slip, epoxy resin-saturated glass roving

## Abstract

This paper presents the findings of an experimental study on the structural response of glass fiber-reinforced polymer (GFRP)-concrete composite beams. The connectors were fabricated from GFRP dowels, epoxy resin-saturated E-glass roving, and/or adhesive layers. The composite beams were subjected to a four-point bending test configuration and examined for their failure modes and load-deformation characteristics. The test results showed that the developed configurations of composite beams significantly outperformed the response of the standalone GFRP I-section profile and non-composite beams. The provision of a discrete interfacial connection successfully prevented the local and lateral torsional buckling of the profile, doubled the initial stiffness, increased the load-carrying capacity by around three times, and imparted a certain degree of ductility and reserve capacity to the otherwise brittle system. The failure occurred primarily due to the shearing of the web. Other modes of failure were observed in the form of the cracking/crushing of concrete, delamination of the laminate, and buckling/crushing of the web. The epoxy-bonded composite beams displayed the highest stiffness, while those with 45° inclined dowels exhibited the highest load-carrying capacity. The results were compared against those predicted by the available analytical expressions, and required modifications are suggested.

## 1. Introduction

Introduced in civil works for the strengthening of existing concrete structures, fiber-reinforced polymer (FRP) laminates are now being recognized as thin-walled load-resisting structural elements [1]. FRP materials are characterized by their high tensile strength, high corrosion resistance, durability in adverse environments, ability to be molded to a variety of shapes and sizes, and low self-weight, providing an ease in transportation and the installation of structural units [2,3,4,5]. The high strength-to-weight ratio of structural members fabricated from FRP laminates significantly reduces the overall self-weight of the structure, leading to a decrease in the foundation dimensions, thereby offsetting the high initial manufacturing cost. However, when provided as thin-walled structural elements, these laminates suffer from discrepancies like a brittle material response, low elastic and shear modulus, and lateral instability. Due to this, the design is generally governed by the deflection and instability criteria [6,7,8], and the material’s strength remains underutilized. To address these shortcomings, hybrid FRP-concrete composite beam systems have been proposed and investigated by past researchers [1,4,5,6,7,8,9,10,11,12,13,14,15,16,17,18,19,20,21,22,23,24,25,26,27,28,29,30,31,32,33,34,35,36]. In one such system, the composite beam assembly comprises an FRP profile with its compression flange either mechanically or adhesively connected to a concrete slab to obtain the T-beam action effect. The concrete slab, acting in conjunction with the FRP profile, resists compressive loads, enhances the overall stiffness of the beam, and provides resistance against local and lateral instability. The FRP web and its tension flange resist the vertical shear and tensile stresses, respectively. However, the structural behavior of these hybrid beams is a complex function of material constitutive laws, interfacial shear stresses, connecting media, span-to-depth ratio, and boundary conditions [37].

In the last three decades, several researchers have tried to develop different prototypes of FRP-concrete composite beams and examine their flexure response. Saiidi et al. [4] observed that the epoxy-bonded box/I-shaped graphite/epoxy-concrete composite beams are susceptible to shear failure of the web, and their stiffness varies proportionally with the compressive strength of concrete. Deskovic et al. [6] concluded that combining steel bolts and epoxy resin and bonding a thin carbon fiber-reinforced polymer (CFRP) laminate to the tension flange imparts a certain degree of pseudo-ductility to the composite beam. Canning et al. [11] proposed that the most practical way of bonding the two components of a hybrid beam is by applying an epoxy-based adhesive to the wet concrete surface. Ribeiro et al. [12] concluded that, as the section width is reduced, the failure mode of an epoxy-bonded U-shaped composite beam shifts from the loss of bond and crushing of concrete to the rupture of the tension flange. Hulatt et al. [13,14] observed that, on varying the number of glass fiber-reinforced polymer (GFRP) layers in the web, the failure mode shifts from shear buckling of web panels to the crushing of concrete. Nordine and Taljsten [1] concluded that connecting a high-strength concrete slab over the compression flange of an I-shaped GFRP beam triples the flexure stiffness and eliminates the possibility of lateral torsional buckling. Li et al. [15] observed that, in a four-point bending test, the I-shaped GFRP-concrete composite deck assembly suffers from a sudden brittle mode of failure in the form of shearing of the web. Correia et al. [7,16] observed that the I-shaped composite beams with bolted connections fail by the shearing of the web at the mid-depth region, whereas the relatively stiffer epoxy-bonded beams suffer from a brittle failure mode at lower loads due to the loss of composite action.

Fam and Skutezky [17] deployed sand-coated GFRP dowels as shear connectors over hollow/concrete-filled box-shaped GFRP profiles. In beams with a hollow profile, failure occurred due to a combined shearing and bending of dowels, whereas, in concrete-filled beams, the tension flange ruptured at failure. Neto et al. [18] and Pinto et al. [19], from their investigation of the flexural response of a footbridge deck consisting of a fiber-reinforced concrete slab bonded to I-shaped GFRP beams, recommended that, for utilizing the full capacity of the developed system, the premature failure of the interfacial connection and the crushing of the web at support points need to be prevented. El-Hacha and Chen [20] and Iskander et al. [21] inferred that, in composite beams with a box-shaped GFRP profile, web-flange junctions are the weakest link, and, irrespective of the flange dimensions, failure occurs due to the shearing of the web at the highly stressed corners. With epoxy bond layer and/or straight steel bolted interfacial connections, Nguyen et al. [22,23] observed a simultaneous crushing of the concrete slab at loading points and rupture of the tension flange at the pure bending moment region. On providing inclined bolts, the beams displayed signs of ductility in the form of extensive cracking of the concrete surrounding the dowel and large interfacial slip. Neagoe et al. [8,24] observed that the I-shaped composite beams with a staggered arrangement of steel bolts display a significant ductile response in the form of the crushing of concrete followed by the sudden delamination of the web-flange junction. Koaik et al. [25] and Jurkiewiez et al. [26] suggested that, to prevent the premature failure of the composite beam with a combined connection of steel bolts and epoxy bond, the shear strength of the bolts should be greater than the debonding load, and the web needs to be strengthened at the support sections. Yang et al. [27], from their study on composite beams with an inverted T-shaped shear key connector, concluded that, in a GFRP-concrete composite beam, as the shear span-to-overall depth ratio increases, stiffness decreases, and the failure mode shifts from the shear failure of concrete to the rupture of the tension flange. Zou et al. [28] tested composite beams with a GFRP C-shaped shear key connector/stay-in-place formwork and concluded that the design of such hybrid beams should be based on the shear capacity of the composite section. Zhang et al. [29,30] observed that, on reinforcing the web in the shear span with carbon fiber laminates, the shear capacity of the composite section becomes significantly enhanced, and the failure mode shifts from the shearing of the web to the rupture of the bottom web-flange junction. In another investigation by the same author [31], ultra-high-performance concrete pockets were used to encase the steel studs and connect the prefabricated concrete slab. The test results showed that beams with continuous concrete pockets perform better than those with discrete pockets.

Ge et al. [32] observed that, in shear key-based composite beams, the failure mode is governed by the section depth, and the bending resistance varies proportionally with the mechanical characteristics of concrete and the profile. Ali et al. [33] observed that the bearing capacity of thin corrugated metal sheets and steel bolt connector-based composite beams is significantly influenced by the presence of web stiffeners, the compressive strength of concrete, and the presence of additional adhesive layers at the interface. Zhang et al. [34], from their investigations on epoxy-bonded GFRP L-section shear key connector-based composite beams, observed that, upon increasing the width of the concrete section, the shear-lag effect starts to significantly influence the overall response of the composite beam. Zhang et al. [35] examined the response of a novel FRP-concrete double-skin tubular composite beam section. The authors observed that the fiber orientation in the outer tube had little or no influence on the overall ductility of the composite beam; however, the concrete crack resistance and bending strength characteristics were found to be the maximum for the one with a ±45° fiber orientation. Recently, for potential applications in large-span bridges, Xue et al. [36] developed a novel externally prestressed FRP-concrete hybrid beam system. The bending test results showed that, depending on the degree of interfacial connection, the developed composite beam configurations ultimately fail either in the form of the shear failure of the profile web at the top web-flange junction or the failure of the interfacial connection.

From the analysis of the reported studies, it can be deduced that most of the earlier research has primarily relied upon steel bolts/studs, epoxy bond layers, shear keys in the form of C/I/inverted-T sections, or a typical combination of these for transferring the interfacial shear stresses. Using steel to fabricate the shear studs and reinforcement in the slab raises questions regarding the applicability of the proposed system in corrosive environments and those requiring electromagnetic shielding. Studies concerning the response of composite beams with GFRP rebars as reinforcement in slab and GFRP dowels as shear studs are not well investigated, particularly in terms of the effect of the angle of inclination of the dowel with the longitudinal axis, and methods of connecting them over the specimen’s compression flange. Also, the response of composite beams with a joint connection of the GFRP dowel and epoxy bond layer provided over a wet concrete surface has not been examined. In addition, almost all previous investigations have considered a high L/D ratio (7.5–10.5); observations regarding the response of composite beams with an L/D ratio of less than five in four-point loading tests are not well documented.

The present study aims at addressing these shortcomings by developing a complete steel-free configuration of GFRP-concrete composite beams that relies on a discrete connection of GFRP dowels (at varied angles of inclination), epoxy bond (over a wet/dried concrete surface), or a typical combination of them for transferring the interfacial shear. To study the influence of shear deformations, the composite beams were fabricated with a low L/D ratio of ~5, and, using repeated wraps of epoxy resin-saturated glass roving, an innovative method of plugging the dowels into the compression flange was proposed. A total of six different configurations of composite beams were developed and tested in a four-point loading test, a relative comparison of the flexure response of the tested beams was made, and their efficacy was established against that of a standalone GFRP profile subjected to the same test conditions. The experimental results were compared against those predicted by the available analytical expressions, and required modifications are suggested. Also, to examine the influence of different structural parameters on the structural response and failure characteristics of the composite beams, a three-dimensional nonlinear analysis, followed by an extensive numerical parametric study, was carried out by the authors using the FEM-based commercial software package *Abaqus/CAE*, the details and findings of which are discussed elsewhere [37].

## 2. Materials and Methods

### 2.1. Specimen Configuration

The test specimens were grouped into three categories (Table 1): (i) Control beam (CB)—standalone GFRP I-section beams; (ii) Non-composite beam (NBC)—no interfacial connection (other than the natural bond) exists between the compression flange of the GFRP profile and the concrete slab; and (iii) Composite beam—discrete interfacial connection either in the form of mechanical connectors or/and adhesive layer is provided between the compression flange of the GFRP profile and the concrete slab. Based on the type of connecting media, the composite beams were further sub-categorized as follows: (a) beams with a straight dowel connection (SDC)—roving tied uniformly spaced (100 mm c/c) double row of GFRP studs as shear connectors; (b) beams with 45° inclined dowel connection (IDC)—roving tied uniformly spaced (100 mm c/c) double row of GFRP studs inclined at an angle of 45° with the beam’s longitudinal axis as shear connectors; (c) beams with epoxy bond connection (EBC)—epoxy-based adhesive layer (~0.50 mm thick) applied over the hardened concrete surface to transfer the interfacial shear; (d) beams with a combined connection of epoxy-based adhesive layer applied over the unhardened concrete surface and roving tied uniformly spaced double row of GFRP studs (EBSDC); and (e) beams with a combined connection of epoxy-based adhesive layer applied over the unhardened concrete surface and roving tied uniformly spaced double row of 45° inclined GFRP studs (EBIDC).

**Figure 1:**
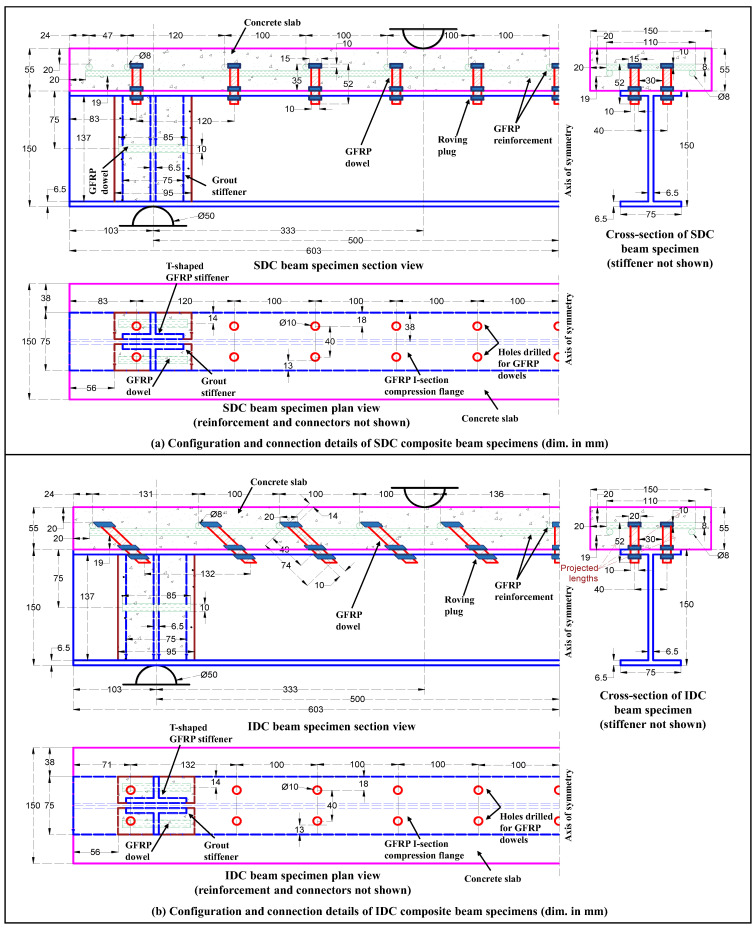
Configuration and connection details of (**a**) SDC composite beam specimens and (**b**) IDC composite beam specimens.

Each specimen type consisted of a 1.206 m long and 150 mm deep pultruded GFRP I-section profile having a flange width of 75 mm and a uniform laminate thickness of 6.5 mm. The concrete slab (precast in EBC specimens and cast in situ in others) had a cross-sectional dimension (width × depth) of 150 × 55 mm and was nominally reinforced with 8 mm diameter pultruded GFRP ribbed rebars to confine the connectors, strengthen the concrete prism, and resist accidental loads during transportation/handling. The composite beams with an overall depth (D) of 205 mm had an effective span (L) of 1 m (103 mm overhang on each side) and were subjected to concentrated loads at each 1/3rd span length, resulting in an L/D ratio of ~5 and a shear span ($l_{s}$)-to-overall depth ratio of ~1.6. The slab width (150 mm) was kept much less than L/4 (=250 mm) to minimize the influence of the shear lag effect, and the slab depth (55 mm) was taken slightly greater than L/20 (=50 mm) to satisfy the nominal cover requirements and fulfill the typical defection criterion of one-way simply-supported concrete slabs [22,38]. The mechanical connections were designed and detailed as per EN 1994-1-1:2004 (E) [39] recommendations for shear connectors, and the bolt hole clearance requirements for FRP bolted connections, as given in ASCE/SEI 74-23 [40]. The mechanical characteristics, load transfer mechanism, and failure modes of the different connection systems, as determined by the authors through an extensive push-out testing regime, are discussed elsewhere [41]. The variability in test results was considered by testing a minimum of two identical specimens of each configuration type (test series#1/#2). Figure 1 illustrates the configuration and connection details of the tested SDC and IDC composite beam specimens (left half portion).

### 2.2. Materials Used

The GFRP I-sections used in the study were machined from the off-the-shelf pultruded profiles. The laminate with a symmetric lamination scheme was fabricated from polyester resin-saturated E-glass roving and chopped glass strand mats. The reinforcement of the concrete slabs and the dowels provided as shear studs were fabricated from off-the-shelf pultruded GFRP ribbed rebars of 8 mm and 10 mm nominal/designated diameter, respectively. The rebars were manufactured from longitudinally oriented vinyl ester resin-saturated E-glass roving bundles. The physical and mechanical characteristics of the pultruded laminate and GFRP rebars/dowels determined from laboratory testing [42,43,44,45,46,47,48] are listed in Table 2. The GFRP profile satisfied the respective minimum strength and stiffness requirements of the E17 grade reinforced plastic composite structural profiles, as specified in the European standard EN 13706-3:2002 [49].

Epoxy resin-saturated E-glass roving in its uncured state was used for tying the joints of the reinforcement provided in the concrete slab and fixing the dowels over the compression flanges of the I-shaped profile. The 1200 TEX (gm/km), 5 mm wide, and 0.17 mm thick E-glass roving had a tensile strength of 314 MPa, which, upon saturating with epoxy resin and subsequent curing (~15 days), was reduced to 267.6 MPa. Based on the type of application, two different versions of epoxy-based adhesives were used in the study. The first kind was a low-viscosity thermosetting matrix used for saturating the E-glass roving and consisted of a clear liquid epoxy resin and a reactive polyamide curing agent. In its cured state, this resin had a tensile strength of 34.4 MPa and an elastic modulus of 1.1 GPa. The second type of adhesive, commercially available with the trade name *Sikadur^®^-330 IN*, was used for bonding the concrete slab to the compression flange of the GFRP profile. The adhesive, exclusively formulated for structural strengthening applications, had a pot life of around 25 min and, upon curing, possessed a tensile strength of 30 MPa and an elastic modulus of 3.5 GPa.

The concrete used in the slab was a normal-density, normal-strength concrete, designed for a 28-day target compressive cube strength of 38.3 MPa. The concrete mix [50] was prepared from 43-grade OPC cement (specific gravity of 3.2), crushed angular-type coarse aggregates (maximum nominal size 10 mm), and finely crushed stone sand (grade zone-II [51]) in a drum mixture at a free water to cement ratio of 0.45. Upon testing [52], the 28-day hardened concrete was found to possess a compressive cube strength of 36.3 MPa, a split tensile strength of 2.3 MPa, an elastic modulus of 30.1 GPa, and a modulus of rupture of 5.1 MPa. To prevent the premature failure of the GFRP profile at the support points, the profile’s web was stiffened with epoxy-bonded T-shaped stiffeners (machined from the flanges of an identical GFRP I-section profile), and the assembly was encased in columns of expansive grout *SikaGrout^®^-214 IN S* (28-day compressive strength of 65 MPa) (Figure 1).

**Table 2:** Properties of pultruded GFRP I-section laminate and rebars/dowels.

Property ^#^	GFRP I-Section Laminate	GFRP Rebars	
		ϕ8 *	ϕ10 *
Fiber volume fraction, $V_{f}$	0.59	0.65	0.65
Mass density, ρ (gm/cc)	2.02	2.04	2.04
Effective diameter, d (mm)	^	8.00	9.50
Longitudinal tensile strength, $\sigma_{1tu}$ (MPa)	324.61	1060.17	1047.94
Longitudinal elastic tension modulus, $E_{1t}$ (GPa)	17.66	20.44	17.74
Longitudinal compressive strength, $\sigma_{1cu}$ (MPa)	324.10	444.53	338.84
Longitudinal elastic compression modulus, $E_{1c}$ (GPa)	11.08	^^^	^^^
Transverse compressive strength, $\sigma_{2cu}$ (MPa)	68.43	^^^	^^^
In-plane shear strength, $\tau_{12}$ (MPa)	48.41	52.91	56.46
Transverse shear strength, $\tau_{13}$ (MPa)	30.23	159.16	164.71
Interlaminar shear strength ^$^, $\tau_{31}$ (MPa)	30.23	50.81	50.33
Longitudinal flexural strength, $\sigma_{1fu}$ (MPa)	412.43	^^^	^^^
Longitudinal elastic flexural modulus, $E_{1f}$ (GPa)	23.18	^^^	^^^
In-plane shear modulus ^&^, $G_{12}$ (GPa)	2.32	^^^	^^^

^#^ Subscripts ‘1’, ‘2’, and ‘3’ represent the longitudinal direction (fiber direction (0°)), in-plane transverse direction (90°), and out-of-the-plane transverse direction (90°) of the laminate, respectively. ^^^ Either the property is not applicable/not experimentally determined in the present study or the required sample size is not available (as with the transverse tensile strength and modulus of the laminate). ^$^ $\tau_{31}\neq \tau_{13}$, as in the case of GFRP rebars; the former is governed by the failure of the matrix and the latter by the shearing of the fibers; for the GFRP I-section laminate, the transverse shear strength is assumed as equal to the interlaminar shear strength. ^&^ Assumed as 1/10th of the corresponding elastic flexural modulus [53]. * Designated diameter of the rebar in mm.

### 2.3. Fabrication of Specimens

The test specimens with straight/45° inclined dowels were fabricated in five stages: Stage #1—marking and machining of GFRP beam, rebars, and dowels to their required lengths; Stage #2—marking, drilling, and finishing of holes in the compression flange of the I beam (in the IDC specimens, the drilled holes were filed to an inclination of 45° with the longitudinal axis of the profile); Stage #3—positioning and riveting of dowels into the pre-drilled holes through repeated wounds (~15 wraps) of epoxy resin-saturated glass roving, applied at the dowel laminate junction on either side of the flange; the roving plug (~20 wraps) was also provided at the dowel end to form a stud head that resists the uplift forces and interlocks the surrounding concrete; Stage #4—strengthening of the profile’s web at the support points using epoxy-bonded T-shaped GFRP stiffeners and grout columns; Stage #5—fabrication of slab reinforcement and casting of concrete slab. Under ambient temperature conditions (~30 °C), the epoxy resin-saturated glass roving required a minimum of 15 days of undisturbed curing.

**Figure 2:**
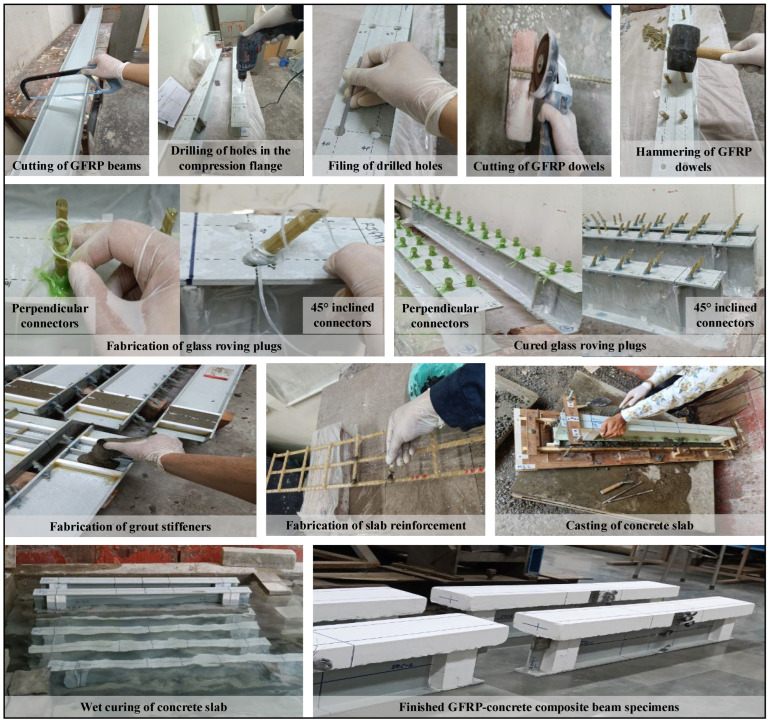
Typical steps followed in the fabrication of composite beam specimens.

For the epoxy-bonded test specimens, first, the concrete slab was cast and allowed to cure for a minimum of 28 days (at 30° C and 55% relative humidity conditions), followed by which the to-be-bonded surfaces of the GFRP beam and concrete slab were sanded and subsequently cleaned with acetone. A 0.50 mm thick layer of uncured *Sikadur^®^-330 IN* adhesive was applied over the prepared surfaces, which were then pressed together under the slab’s weight and left undisturbed for a minimum of 24 h. In specimens where a combination of dowels and an epoxy layer was provided, the compression flange with rigidly attached shear studs (i.e., fully cured roving plugs) was first coated with a 0.50 mm thick layer of uncured *Sikadur^®^-330 IN* adhesive, over which the fresh concrete was poured and compacted. Since the concrete slabs were cast in separate batches, to account for the variability and obtain the exact value of their compressive strength at the time of testing of the composite beams corresponding to each batch, 150 mm standard cubes were cast. The average compressive strength of these cubes on the day of testing was around 41.50 MPa (at a coefficient of variation of 0.045), and the elastic modulus was around 31.62 GPa. The various steps followed in the fabrication of specimens are shown in Figure 2.

### 2.4. Test Setup and Instrumentation

**Figure 3:**
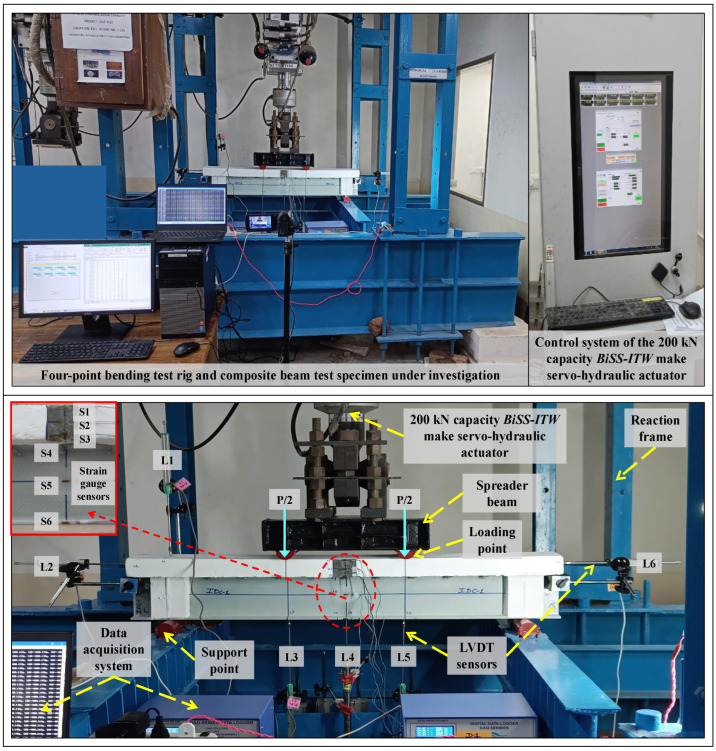
Test setup and instrumentation of the composite beam specimen.

The flexural response of the specimens was examined by subjecting them to a four-point loading configuration. The test setup and specimen instrumentation details are shown in Figure 3. The beams were kept simply-supported over 125 mm long, 50 mm dia. mild steel roller supports and loaded at each 1/3rd span length sections through 200 mm long, 50 mm dia. semi-circular mild steel rollers (welded to a spreader beam). The test was conducted in a displacement-controlled mode at a rate of 2 mm/min. The load (P) was applied through a 200 kN load capacity *BiSS-ITW* make servo-hydraulic actuator and was measured through in-built load cells. A total of six 100 mm stroke length LVDT sensors were deployed to measure the corresponding vertical displacement at mid (L4) and 1/3rd span sections (L3,L5), interfacial slip at beam ends (L2,L6), and uplift (L1). The normal/longitudinal strains at the mid-span section were monitored using a set of six 10 mm long strain gauges attached along the section’s depth at critical points (S1: concrete top, S2: concrete mid, S3: concrete bottom, S4: GFRP top flange, S5: GFRP mid-web, S6: GFRP bottom flange). The gauged variables were recorded at a frequency of 3 Hz by a 2370MS high-speed data logger unit and transferred to a digital computer for analysis.

## 3. Results and Discussions

### 3.1. Flexural Response and Failure Modes

The flexural response and the associated failure characteristics of each of the tested beam specimens are examined from the load-deformation curves, vertical deflection profile plots at different load levels, variation of normal strains at critical points lying along the depth of the mid-span section with the increase in load, normal strain profile of the mid-span section at different load levels and the associated shift in the neutral axis (N.A.), load-vs.-end slip plots, and the failure mode(s). Table 3 summarizes the mechanical characteristics (average of the two identical test specimens (magnitude in the case of interfacial slip) (series #1 and series #2)) and the corresponding primary and ultimate mode(s) of failure of the different beam configurations examined in the present study; the inferences made from the analysis of the same are discussed in the following sections.

#### 3.1.1. Control Beam (CB)

**Figure 4:**
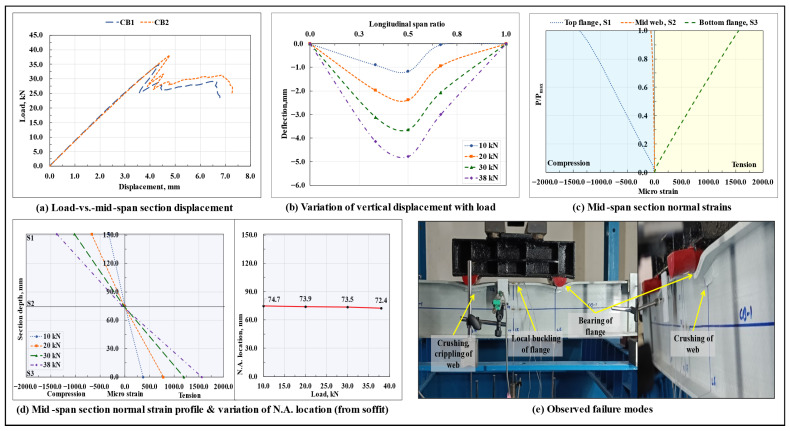
Flexural response and failure modes observed in the tested CB specimens.

The load-displacement response of the standalone GFRP I-section beams is shown in Figure 4a, and the variation of vertical displacement along span length at different load levels in the CB2 specimen is shown in Figure 4b. Resulting from the linear elastic behavior of FRP materials, both the tested control beam specimens exhibited a linear elastic response till the peak load $\left(P_{max}\right)$. The failure initiated in the form of the delamination of the web-flange junction under one of the point loads, followed by a sudden subsidence of this section (as caused by the crippling of the web). As the load was increased further, the web-flange junction under both the loading points got delaminated, followed by which the local buckling of the compression flange occurred in the bending span; bearing failure/deformation/delamination of the top flange occurred under the loading points, and the crushing of the web occurred at the loaded sections. At/beyond the peak load, arising from the local buckling of the compression flange, the portion of the beam spanning in the pure bending moment region attained an upward convexity, thereby causing a sudden reversal of the vertical displacement (as can be observed in the corresponding load-displacement curves at/beyond the peak load). The variation of normal strains with load and their distribution across the depth of the highly stressed mid-span section at different load levels for the CB2 specimen are shown in Figure 4c and Figure 4d, respectively. Till the peak load, the normal strains in the mid-web and bottom flange regions varied linearly with load, whereas, in the compression flange, due to the buckling of the flange close to the peak load (Figure 4e), the normal strains started varying nonlinearly with load.

Throughout the test, the top flange and the upper half of the web region were under compression, the lower half of the web region and the bottom flange were under tension, and the neutral axis (N.A.) remained close to the centroidal axis level of the GFRP I-section. As no signs of rupture/crushing/delamination of the GFRP laminate in either tension or compression at the maximum bending moment mid-span section were observed at failure, the maximum normal strain values attained at the highly stressed extreme fibers of the GFRP I-section at this section remained well below their respective ultimate values. Also, as the local buckling of the compression flange, bearing failure of the top flange under the loading points, and subsequent crushing of the web at these locations preceded the lateral-torsional buckling of the beam, a very marginal amount of lateral deflection (gauged at the top compression flange) at the mid-span and end span sections of the beam was observed till failure.

#### 3.1.2. Non-Composite Beam (NBC)

**Figure 5:**
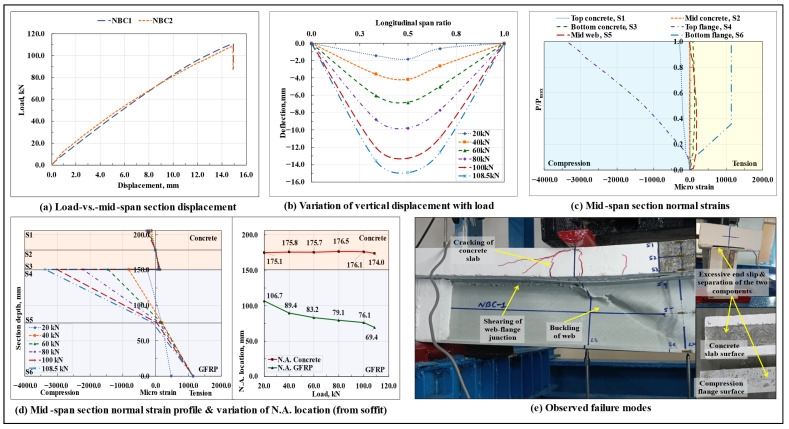
Flexural response and failure modes observed in the tested NBC specimens.

The load-displacement response of the non-composite beams is shown in Figure 5a, and the variation of vertical displacement along the span length at different load levels in the NBC2 specimen is shown in Figure 5b. The progressive cracking of the concrete slab under the loading points led to a slight nonlinearity in the corresponding load-displacement response. On average, the load-carrying capacity $\left(P_{max}\right)$ of the non-composite beams was found to be almost thrice that of the standalone GFRP I-section beams. However, the initial stiffness (secant stiffness at ${0.6P}_{max}$ load level) of the NBC specimens remained close to that of the standalone GFRP profile (resulting from the absence of a discrete interfacial shear connection, the two components (GFRP I-section profile and concrete slab) spanned individually, and, due to its relatively high deformability, the GFRP I-section profile component dominated the overall response).

The failure initiated in the form of the loss of the interfacial bond (natural bond between the GFRP-concrete surfaces, as indicated by the generation of interfacial slip at the beam ends), followed by which a progressive development of flexure and shear cracks started to occur in the concrete slab in the pure bending moment and shear regions, respectively, that continued till the peak load. The development of cracks in the concrete slab and noticeable slip at the beam ends imparted a certain level of ductility to the beams. The beams ultimately failed in a sudden catastrophic manner due to the longitudinal (0°, local principal material axis 1 of the profile laminate) in-plane shearing of the web-flange junction of the GFRP I-section profile in either of the shear spans and the simultaneous buckling of the web under one of the loading points.

The load-vs.-strain plots of the NBC2 specimen (Figure 5c,d) show that, following the generation of interfacial slip, two separate neutral axes formed in the beam section, one in the concrete section and the other in the GFRP I-section, thereby indicating a complete loss of the composite action. As the load increased, in the mid-span section, the neutral axes (initially located close to the GFRP-concrete interface) started shifting towards their respective centroidal axis depths. This shows that the naturally occurring GFRP laminate-concrete bond is inadequate in providing the required composite action, and hence, to enable the concrete slab to work in conjunction with the GFRP I-section profile, a discrete shear connection needs to be provided at the interface.

The normal strains developed in the concrete slab varied nonlinearly with load due to the progressive cracking of concrete under the loading points; on the other hand, those developed at the highly compressed top flange of the GFRP I-section varied nonlinearly with load primarily due to the nonlinear response of FRP materials under compressive loads [3,55]. On average, at around 28% of the peak load, the bottom web-flange junction of the GFRP I-section profile at the mid-span section experienced microstructural-level damage, due to which, beyond this, no further strain increment was observed in the tension flange of the GFRP I-section profile in the mid-span section. Since the failure occurred primarily due to longitudinal in-plane shearing of the GFRP I-section profile web-flange junction, the maximum normal strain values attained at the highly stressed extreme fibers of the concrete slab and the GFRP I-section at the mid-span section did not reach their respective ultimate values. At peak load, both beams, on average, sustained an interfacial slip of around 3.38 mm at the right end and 2.92 mm at the left end. The observed failure modes are shown in Figure 5e.

#### 3.1.3. Straight Dowel Connection Based Composite Beam (SDC)

**Figure 6:**
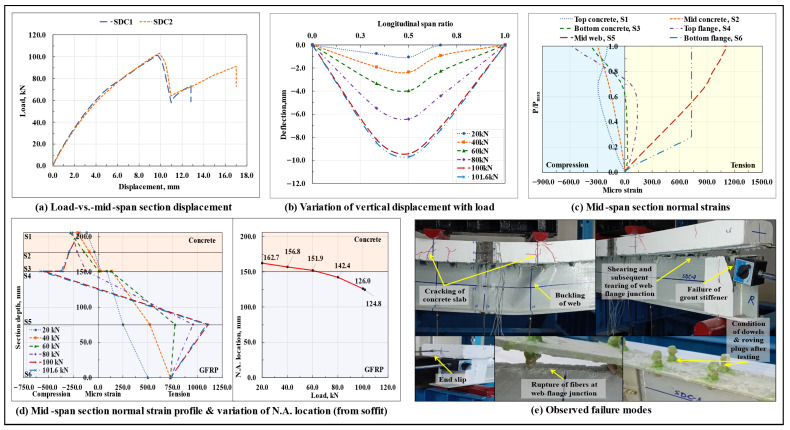
Flexural response and failure modes observed in the tested SDC specimens.

The load-displacement response of the composite beams with straight dowel connectors is shown in Figure 6a, and the variation of vertical displacement along span length at different load levels in the SDC1 specimen is shown in Figure 6b. The SDC composite beams displayed a bilinear load-displacement response till the peak load $\left(P_{max}\right)$, following which, a gradual drop in load was reported, which was succeeded by a secondary hardening phase that continued till the ultimate failure of the beam. On average, at around ${0.6P}_{max}$ load level, cracks started to appear in the concrete slab under the loading points and in the concrete wedge surrounding the dowels, followed by a sudden increase in the interfacial slip (which was almost negligible till this load level) at the end regions; this caused a shift in the characteristics of the interfacial connection from being a full/rigid connection (no-slip condition) to a partial/flexible connection, and led to a sudden drop in the stiffness of the composite beam. From the plots of variation of normal strains at critical points lying along the depth of the mid-span section with the increase in load, the normal strain profile of the mid-span section at different load levels, and the associated shift in the neutral axis (N.A.) of the SDC1 specimen (Figure 6c,d), the presence of a single N.A. in the mid-span section shows that the epoxy resin-saturated glass roving tied GFRP shear studs were successful in imparting the required composite action to the beam assembly till failure. With an increase in load, as resulting from the extensive cracking in the concrete surrounding the dowels, the neutral axis, initially located in the concrete slab close to the GFRP-concrete interface, started shifting towards the centroidal axis level of the GFRP I-section. The nonlinear variation of normal strains with the depth of the composite section shows a considerable influence of shear deformations in the overall flexure response of the composite beam.

The initiation of failure was indicated by the appearance of flexure and flexure-shear cracks in the concrete slab under the loading points, sounds of progressive cracking of the concrete wedge surrounding the dowels, delamination of the GFRP I-section profile laminate, and formation of the end slip. The composite beam primarily failed due to the longitudinal in-plane shearing of the top web-flange junction of the GFRP I-section profile. Also, the excessive bearing stresses exerted by the end dowels over the upper half of the support stiffener caused the failure of the grout column (Figure 6e). Unlike the non-composite beam specimens, in which the buckling of the web and tearing off of the web-flange junction took place simultaneously, in the straight dowel connection-based composite beam specimens, a certain lag (more prominent in the SDC2 specimen) was observed between the tearing of the web-flange junction and eventual buckling of web under one of the loading points, which also explains the secondary ascending arm observed in the corresponding load-vs.-vertical displacement curves after the drop in the peak load. The lag could have been caused by the rigidity imparted to the section by the composite action. The lag in the occurrence of the ultimate failure imparted a certain level of reserved strength and ductility to the composite beams. In addition, tension/flexure cracks were also observed in the top portion of the concrete slab at the support sections primarily due to the excessive bearing stresses exerted by the end region dowels over the surrounding concrete (at beam ends, the dowels were responsible for resisting the maximum mobilized value of the interfacial slip) and formation of a minor upward convexity at these regions due to the anchorage provided by the stud heads against uplifting of the concrete section at the beam ends.

On average, the load-carrying capacity of the SDC beams was found to be marginally lower (on average, ~6.5% lower) than that of the non-composite beams and almost thrice that of the standalone GFRP I-section beams (though both SDC and NBC beams suffered the same mode of primary and ultimate failure; however, due to the increase in stiffness (which attracted relatively high stresses in the beam), weakening of the compression flange of the GFRP I-section profile due to the drilling of holes, a sudden spike in the horizontal shear stresses in the portion of the top web-flange junction of the GFRP I-section profile lying between the two dowel containing sections (at which a certain portion of these stresses was relieved by the dowels), along with the concentration of stresses in the concrete prism surrounding the dowels that led to excessive cracking in the concrete wedge surrounding the dowel, altogether marginally reduced the load-carrying capacity of the composite beam); however, a significant enhancement in stiffness at the service load level was observed (secant stiffness at ${0.6P}_{max}$ load level roughly corresponded to the typical service load deflection limit of L/250 [54]), where the stiffness of the non-composite beam specimens was found to be close to that of the standalone GFRP I-section beams; the initial stiffness of the SDC composite beams was observed to be around 73% higher than that of the standalone GFRP I-section beams, on average. The normal strains in the concrete slab and the top flange of the GFRP I-section varied nonlinearly with load due to the cracking of the concrete under the loading points and shifting of the N.A. towards the mid-height of the I-section (which brought the top flange of the GFRP I-section under compression), respectively.

On average, at around 36% of the peak load, the bottom web-flange junction of the GFRP I-section profile at the mid-span section ruptured at the microstructure level, due to which no further strain increment was observed in the bottommost tension flange, followed by which the majority of the tensile stresses in the mid-span section were resisted by the portion of the web lying below the N.A. At peak load, the SDC composite beams, on average, sustained an interfacial slip of around 1.77 mm at the right end and 1.97 mm at the left end, which were respectively ~47.6% and ~32.5% lower than the end slips experienced by the non-composite beams. Following the testing procedure and removal of the surrounding concrete, upon inspecting the state of the connectors, no dispositioning of the dowels and no visual signs of damage in the form of the rupture/delamination of dowels and/or detachment/debonding/rupture of the epoxy resin-saturated glass roving plugs were observed.

#### 3.1.4. 45° Inclined Dowel Connection Based Composite Beam (IDC)

**Figure 7:**
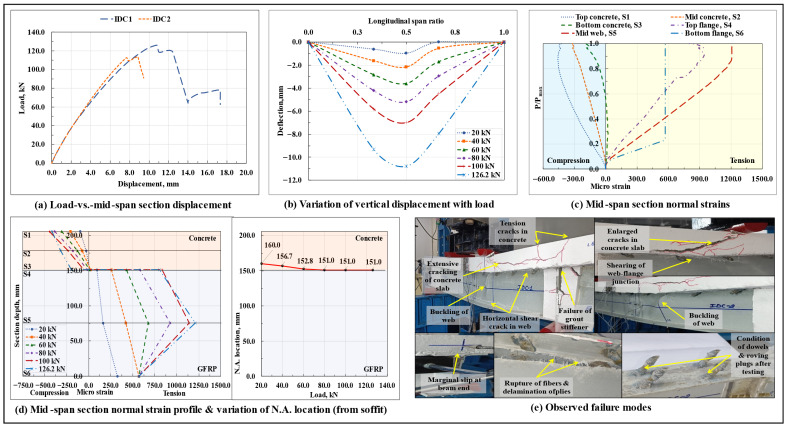
Flexural response and failure modes observed in the tested IDC specimens.

The load-displacement response of the composite beams with 45° inclined dowel connectors is shown in Figure 7a, and the variation of vertical displacement along the span length at different load levels in the IDC1 specimen is shown in Figure 7b. The IDC composite beams displayed a nonlinear load-displacement response till the peak load $\left(P_{max}\right)$. In one of the tested specimens (IDC1), following the drop in the peak load, a secondary rise in load that continued till the ultimate failure of the beam was also observed. Compared to the non-composite beams, a very marginal amount of interfacial slip (initiated at around ${0.6P}_{max}$ load level) was observed at the IDC beam ends. At peak load, these composite beams, on average, sustained a marginal interfacial slip of around 0.82 mm at the right end and 0.54 mm at the left end, which were respectively ~75.7% and ~81.5% lower than the end slips experienced by their non-composite beam counterparts. From the plots of variation of normal strains at critical points lying along the depth of the mid-span section with the increase in load, normal strain profile of the mid-span section at different load levels, and the associated shift in the neutral axis (N.A.) of the IDC1 specimen (Figure 7c,d), it can be observed that till the peak load, the concrete slab was primarily subjected to compressive stresses, and the tensile stresses were resisted by the GFRP I-section profile. At the start of the test, the N.A. of the mid-span composite section was present in the concrete slab, and, with the increase in load, it shifted towards the GFRP-concrete interface. As the N.A. maintained its position at/close to the GFRP-concrete interface throughout the test, it can be concluded that the composite action exhibited by the 45° inclined dowel connection-based composite beam specimens was close to that of an ideal composite beam.

The significant differences observed in the behavior of the load-displacement curves of the identical IDC1 and IDC2 specimens can be attributed to the dissimilarity in their exhibited failure modes. In the IDC1 specimen, the failure initiated in the form of the formation of flexure and flexure-shear cracks in the concrete slab under the loading points, which was accompanied by the progressive cracking of the concrete wedge surrounding the dowels and delamination of the GFRP I-section profile laminate, followed by which a horizontal shear crack appeared in the web of the GFRP I-section profile close to the bottom web-flange junction; the crack started from the right end support of the beam and extended towards the mid-span section. The composite beam specimen experienced a sudden longitudinal in-plane shearing of the top web-flange junction of the GFRP I-section profile, which was then followed by the buckling of the web under one of the loading points (occurred after a certain lag, thereby leading to the formation of a secondary ascending arm in the corresponding load-vs.-vertical displacement curve after the drop in the peak load), the formation of extensive shear cracks in the concrete slab, and the failure of the grout column (Figure 7e). On the contrary, in the IDC2 specimen, after the formation of initial cracks in the concrete section and the interlaminar delamination of the GFRP I-section profile laminate, no discrete horizontal shear crack appeared in the profile web near the bottom web-flange junction; instead, close to the peak load, a horizontal in-plane shear crack appeared in the right shear span region at the top web-flange junction of the GFRP I-section profile (causing a sudden dip in the corresponding load-vs-vertical displacement curve), which, with the increase in load, elongated and sheared off the top web-flange junction of the GFRP I-section profile. The action was instantaneously accompanied by the buckling of the web and excessive crushing of the concrete slab under one of the loading points. The differences/discrepancies in the composite microstructure, properties of concrete surrounding the dowel, degree of compaction of concrete, and marginal variation in the structural layout of the 45° inclined dowels due to fabrication errors/defects, etc., could be the possible reasons behind such a divergence in the behavior of the two identical specimens.

Similar to the straight dowel connection-based composite beams, pertaining to the previously stated reasons, in the IDC composite beam specimens also, tension/flexure cracks were formed in the top portion of the concrete slab at the support sections, and no visible signs of damage in the connectors and/or in the epoxy resin-saturated glass roving plugs was observed after the test. Unlike its straight dowel connection-based composite beam counterpart, resulting from a relatively better display of composite action, the IDC composite beams, on average, were found to possess a load-carrying capacity that was around 9.56% higher than that of the non-composite beams and almost 3.3 times that of the standalone GFRP I-section beams. On the other hand, the initial stiffness of these beams was also almost twice that of the standalone GFRP I-section beams. Close to the peak load, as resulting from the excessive cracking of concrete, the normal strains in the concrete slab became nonlinear, while those in the GFRP I-section (being subjected primarily to tensile stresses throughout the test) remained linear till the failure of the beam.

On average, at around 39% of the peak load, the bottom web-flange junction of the GFRP I-section profile at the mid-span section suffered microstructural level damage, due to which no further strain increment was observed in the bottommost tension flange, and the top flange and web of the GFRP I-section started to resist the tensile stresses in the mid-span section. The absence of a sudden drop in stiffness (as observed in the SDC composite beam specimens), shows that providing a 45° inclination to the otherwise straight dowels leads to a better anchorage of the dowel with the surrounding concrete, a better distribution of stresses between the dowels and concrete, minimizes the concentration of bearing stress at the dowel-concrete boundary, reduces the extent of premature cracking of the concrete wedge surrounding the dowel, altogether preventing the composite beams from undergoing a sudden drop in stiffness and partial loss of connection rigidity.

#### 3.1.5. Epoxy-Bonded Composite Beam (EBC)

**Figure 8:**
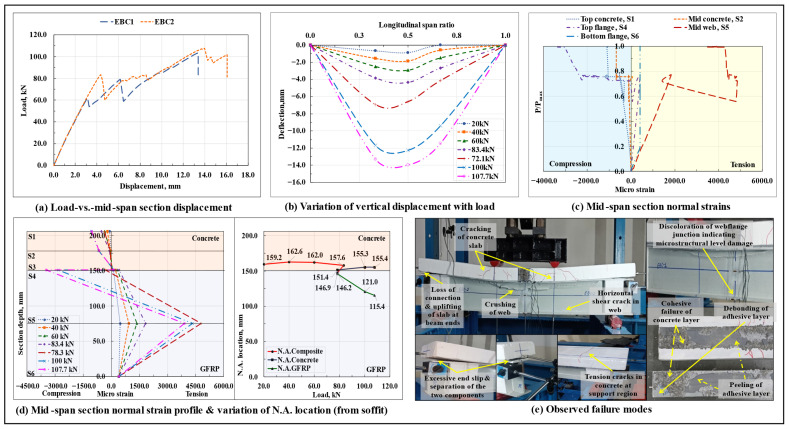
Flexural response and failure modes observed in the tested EBC specimens.

The load-displacement response of the epoxy-bonded composite beams is shown in Figure 8a, and the variation of vertical displacement along span length at different load levels in the EBC2 specimen is shown in Figure 8b. The load-vertical displacement curves followed a linear response till the point of initiation of failure of the interfacial bond, which occurred at around ${0.6P}_{max}$ and ${0.77P}_{max}$ load levels in the EBC1 and EBC2 composite beam specimens, respectively. The dissimilarity in response could have been caused by the differences in the properties of the bonded concrete surface, variations in the thickness of the adhesive layer, and non-uniformity/unevenness in the clamping pressure applied over the adhesive layer during its curing period. Beyond the point of the initiation of debonding, a significant progression of the interfacial slip was observed at the beam ends (which was almost close to zero till this load level). With a further increase in load, a progressive failure of the remaining interfacial bond started to occur, which led to subsequent undulations in the corresponding load-displacement curves till the peak load $\left(P_{max}\right)$. The epoxy-bonded composite beams ultimately failed due to the sudden crushing of the upper half of the GFRP I-section profile web (compared to the shear span regions of the beam, the crushing occurred relatively more extensively in the pure bending moment region) (primarily due to the excessive incompatibility between the curvatures of the two bonded components in the pure bending moment region, as caused by the relatively high stiffness of the bonded composite beam and the individual deformability characteristics of the two bonded components); the action was accompanied by a complete separation (total debonding or a complete loss of composite action at all sections of the beam) of the concrete slab from the bonded flange of the GFRP I-section profile, which exaggerated the interfacial slip value at either of the beam ends. In the mid-span section, as inferred from the plot of the normal strain profile along the section depth at different load levels of the EBC2 specimen (Figure 8d), the loss of composite action occurred way before the crushing of the web (${0.77P}_{max}$ load level); however, at this load level, a complete separation of the two bonded components did not occur. The final separation of the two components occurred instantaneously after the crushing of the web in the EBC1 specimen and was lagged by a certain amount in the EB2 specimen (explaining the undulations and formation of a secondary ascending arm in the corresponding load-displacement curve of the same after the drop in the peak load).

At peak load, the beams, on average, sustained an interfacial slip of around 0.36 mm at the right end and 3.44 mm at the left end. A significant difference in the slip values recorded at the two ends of the beam resulted from the uneven/non-uniform/progressive shearing/peeling/rupture of the adhesive layer that anchored the concrete slab from moving along one of the longitudinal directions and advanced its movement in the other. In the EBC2 specimen, close to the failure load, the crushing of the web was also preceded by the appearance of a horizontal shear crack near the bottom web-flange junction of the GFRP I-section profile in one of the shear span regions. Upon a visual inspection of the failure surfaces after the tests, noticeable signs of non-uniform/uneven/progressive debonding/peeling/shearing of the adhesive layer and cohesive failure of the concrete surface in the vicinity of the bonded interface in the form of damage to the concrete matrix were observed.

From the plots of variation of normal strains at critical points lying along the depth of the mid-span section with the increase in load, normal strain profile of the mid-span section at different load levels, and the associated shift in the neutral axis (N.A.) of the EBC2 specimen (Figure 8c,d), it can be observed that, following the point of initiation of cracking of the concrete slab under the loading points, the mid-span section N.A. that initially resided in the concrete slab as a single bending axis (representing the presence of composite action) started shifting towards the GFRP-concrete interface, and, beyond the point of initiation of debonding, as resulting from the complete loss of the composite action at this section, got divided into two separate components. Following the debonding initiation load level, arising from the occurrence of abrupt and sudden changes at the bonded interface, highly incoherent and haphazard values were recorded by the strain gauges (the reason why the strain variation recorded by the gauge S3 is not shown in Figure 8c).

On average, at around 21% of the peak load, microstructure-level damage occurred at the bottom web-flange junction of the GFRP I-section profile in the mid-span section, which prohibited the further participation of the bottom flange in resisting the tensile stresses at this section. On average, arising from the differences in the ultimate failure mode, the load-carrying capacity of the EBC composite beams was found to be marginally lower (on average, ~3.6% lower) than that of the non-composite beams and nearly 2.9 times that of the standalone GFRP I-section beams; however, resulting from the presence of a relatively rigid and brittle bonded interfacial shear connection, these composite beams demonstrated the highest amount of initial stiffness amongst all of the tested beam configurations; on average, their initial stiffness was found to be around 2.4 times that of the non-composite and standalone GFRP I-section beams. The observed failure modes are shown in Figure 8e.

#### 3.1.6. Epoxy Bond and Straight Dowel Connection Based Composite Beam (EBSDC)

**Figure 9:**
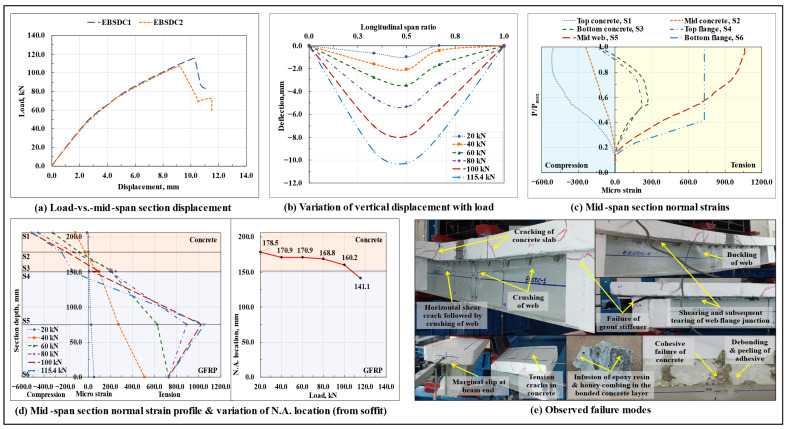
Flexural response and failure modes observed in the tested EBSDC specimens.

The load-displacement response of the epoxy-bonded and straight dowel connected composite beams is shown in Figure 9a, and the variation of vertical displacement along the span length at different load levels in the EBSDC1 specimen is shown in Figure 9b. Similar to their SDC counterparts, the EBSDC composite beam specimens also displayed a bilinear load-displacement response till the peak load $\left(P_{max}\right)$. At around ${0.7P}_{max}$ load level, arising from the high concentration of bearing stresses at the dowel-concrete boundary, extensive cracking occurred in the concrete prisms surrounding the dowels; this led to the generation of excessive interfacial slip and caused a sudden dip in the stiffness of the composite beam. In the tested EBSDC1 specimen, the failure took place in three stages: Stage #1—the appearance of flexure and flexure-shear cracks in the concrete slab under the loading points, accompanied by the sounds of progressive cracking of the concrete section surrounding the dowel and delamination of the GFRP I-section profile laminate; Stage #2—the longitudinal in-plane shearing of the web of the GFRP I-section profile close to the top web-flange junction in the left shear span; and Stage #3—the extension of the developed shear crack over the entire span length, followed by a sudden crushing of the upper half of the GFRP I-section profile web, accompanied by the development of excessive shear cracks in the concrete slab in the left shear span region and tension/flexure cracks in the top portion of the concrete slab at/near support sections.

On the contrary, in the EBSDC2 specimen, the epoxy-based adhesive layer that was applied over the wet concrete surface appeared ineffective, as the failure mode(s) was similar to that of the SDC composite beam specimens, in which, followed by the initial cracking of the concrete slab and delamination of the GFRP I-section profile laminate, longitudinal in-plane shearing of the top web-flange junction of the GFRP I-section profile took place in either of the shear spans, which later being accompanied by the local buckling of the web under one of the loading points and formed a minor secondary ascending arm after the drop in the peak load, as observed in the load-displacement curve of the same. Upon a visual inspection of the failure surfaces after the tests, no noticeable signs of damage/delamination and/or dispositioning of the GFRP studs and of detachment/debonding/rupture of the epoxy-saturated tensioned E-glass roving plugs were observed; the epoxy-bonded surface of the compression flange of the GFRP I-section profile, however, showed some minor signs of debonding/peeling/shearing of the adhesive layer and cohesive failure of the concrete surface in the vicinity of the bonded interface and in and around the dowels (though not as prominent as those observed in the EBC composite beam specimens). The epoxy-based adhesive layer applied over the fresh concrete appears to have integrated with the cement matrix, did not effectively bond with the surface of the GFRP I-section profile laminate, and, upon compaction of the concrete slab, caused honey-combing in the concrete surface in the vicinity of the bonded interface.

From the plots of variation of normal strains at critical points lying along the depth of the mid-span section with the increase in load, normal strain profile of the mid-span section at different load levels, and the associated shift in the neutral axis (N.A.) of the EBSDC1 specimen (Figure 9c,d), the presence of a single neutral axis in the mid-span section throughout the test showed that the provided interfacial shear connection was successful in imparting and maintaining the required composite action till the ultimate failure of the beam. With an increase in load, as arising from the excessive cracking of the concrete section around the dowels and under the loading points, the N.A. started shifting from the mid-height region of the concrete slab towards the top web-flange junction of the GFRP I-section. However, in the second identical EBSDC2 specimen, close to the peak load, as caused by the total separation/shearing off of the top web-flange junction of the GFRP I-section profile, the beam lost its composite action, thereby leading to the formation of separate/individual neutral axes in the two components of the beam. The cracking of the concrete section under the loading points and around the dowels, the shifting of the N.A. towards the centroid level of the GFRP I-section, and the crushing of the web of the GFRP I-section profile, altogether led to a nonlinear variation of normal strains in the corresponding gauge regions. On average, at around 46% of the peak load, microstructure-level damage occurred at the bottom web-flange junction of the GFRP I-section profile in the mid-span section, which prohibited the further participation of the bottom flange in resisting the tensile stresses at this section.

Based on the observed response, however, it cannot be concluded that providing an adhesive layer over the wet concrete surface altogether appeared ineffective, as, relative to their SDC composite beam counterparts, the dual connection-based composite beams caused a difference in the primary failure mode and led to an improvement of around 8.73% in the load-carrying capacity and of around 10.85% in the initial stiffness. In addition, the interfacial slip values at the peak load level also got marginally reduced to 1.53 mm at the right end and 1.56 mm at the left end. The observed failure modes are shown in Figure 9e.

#### 3.1.7. Epoxy Bond and 45° Inclined Dowel Connection Based Composite Beam (EBIDC)

**Figure 10:**
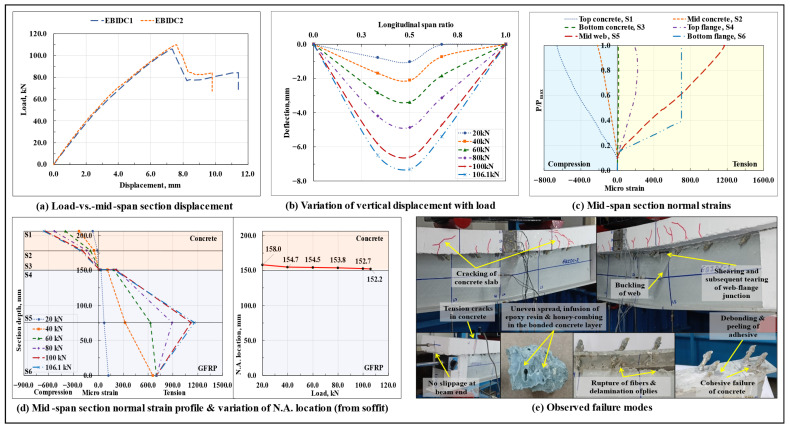
Flexural response and failure modes observed in the tested EBIDC specimens.

The load-displacement response of the epoxy-bonded and 45° inclined dowel connected composite beams is shown in Figure 10a, and the variation of vertical displacement along the span length at different load levels in the EBSDC1 specimen is shown in Figure 10b. As seen previously in the EBSDC composite beam specimens, where the presence of an additional adhesive layer at the interface did not significantly influence the flexural response of the composite system, in the case of the EBIDC composite beam specimens also, the behavior was found to be almost coherent with their unbonded counterparts (IDC composite beam specimens). The composite beams displayed a nonlinear load-displacement response till the peak load $\left(P_{max}\right)$, and no signs of excessive cracking in the concrete prism surrounding the inclined dowel and/or excessive progressive debonding/peeling/shearing of the adhesive layer were observed prior to the primary failure of the beam. Compared to their standalone inclined dowel counterparts (IDC composite beam specimens), the presence of an additional adhesive layer applied at the GFRP-concrete interface over a wet concrete surface enhanced the initial stiffness of the composite beam by about 9.3% and almost eliminated the interfacial slip at the beam ends (slip at the right end got reduced to 0.04 mm and that at the left end to 0.11 mm); however, as arising from an increase in stiffness (which attracted relatively high stresses in the beam) and the marginal difference observed between the primary mode of failure of the two, the load-carrying capacity got reduced by around 10%. Other than that, unlike the IDC composite beam specimens, the presence of secondary connecting media in the form of an adhesive layer at the GFRP-concrete interface in the case of EBIDC composite beams led to a relatively better distribution of shear stresses between the concrete slab and the web of the GFRP I-section profile and prevented the occurrence of extensive shear cracking in the concrete slab after the in-plane shearing and subsequent buckling of the GFRP I-section profile web under the loading points.

As observed previously with the EBC composite beam specimens, in this case also a significant difference in the slip values was observed at the two extremes of the composite beam, which could have been caused by the non-uniform/uneven/progressive debonding/peeling/shearing of the adhesive layer, which anchored the concrete slab from moving along one of the longitudinal directions and advanced its movement in the other. As preceded by the formation of flexure and flexure-shear cracks in the concrete slab, the EBIDC composite beam specimens failed due to the longitudinal in-plane shearing of the top web-flange junction of the GFRP I-section profile in the left shear span region, which was followed by the local buckling of the web of the GFRP I-section profile under one of the loading points (more prominent in the EBIDC1 specimen), which also explains the appearance of a secondary ascending arm after the drop in the peak load, as observed in the corresponding load-displacement curves, which incorporated a certain degree of ductility to the otherwise brittle system. In addition, close to the peak load, tension/flexure cracks also formed at the top surface of the concrete slab at the left support section, and the upper half of the corresponding grout stiffener that was in contact with the end dowels also broke off. Upon a visual inspection of the failure surfaces after the tests, no signs of damage were observed in any of the 45° inclined GFRP studs; also, as previously seen in the EBSDC composite beam specimens, noticeable signs of debonding/peeling/shearing of the adhesive layer, cohesive failure in the bonded concrete surface, infusion of the adhesive with the concrete matrix, and honey-combing in the concrete layer close to the GFRP-concrete interface were visible.

From the plots of variation of normal strains at critical points lying along the depth of the mid-span section with the increase in load, the normal strain profile of the mid-span section at different load levels, and the associated shift in the neutral axis (N.A.) of the EBIDC1 specimen (Figure 10c,d), it can be observed that, similar to its unbonded counterpart (IDC composite beam) and in the EBIDC composite beam specimens also, the mid-span section N.A. remained close to the GFRP-concrete interface throughout the test and imparted an almost ideal composite action to the beam till failure. The relatively lower concentration of bearing stresses in the concrete surrounding the dowels led to a substantial reduction in the concrete microcracking and resulted in an almost linear load-normal strain variation in the concrete slab till failure. At around 38% of the peak load level, due to the microstructure level ply failure in the bottom web-flange junction at the mid-span section, the bottom GFRP flange at this region became ineffective in resisting any further tensile stresses. The observed failure modes are shown in Figure 10e.

### 3.2. Comparison of Response

**Figure 11:**
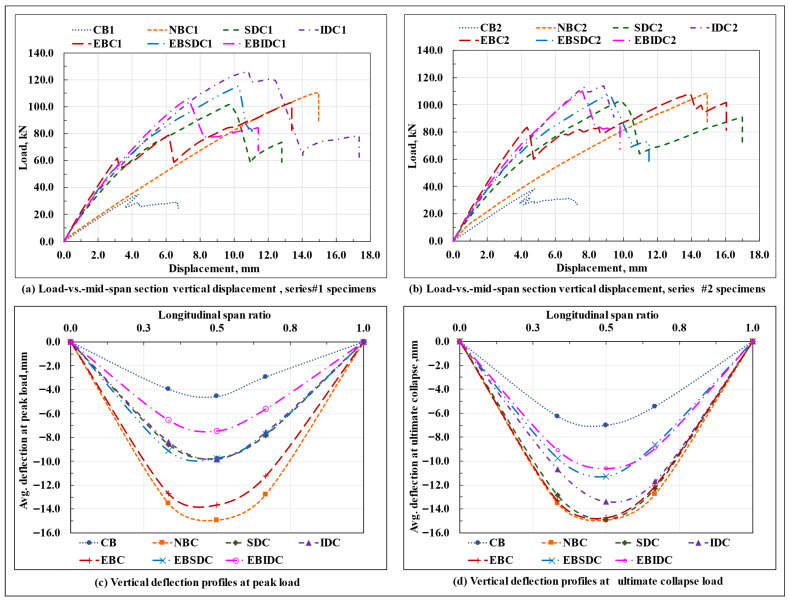
Load-vs.-mid-span section vertical displacement plots; vertical deflection profiles of the tested beam specimens.

**Figure 12:**
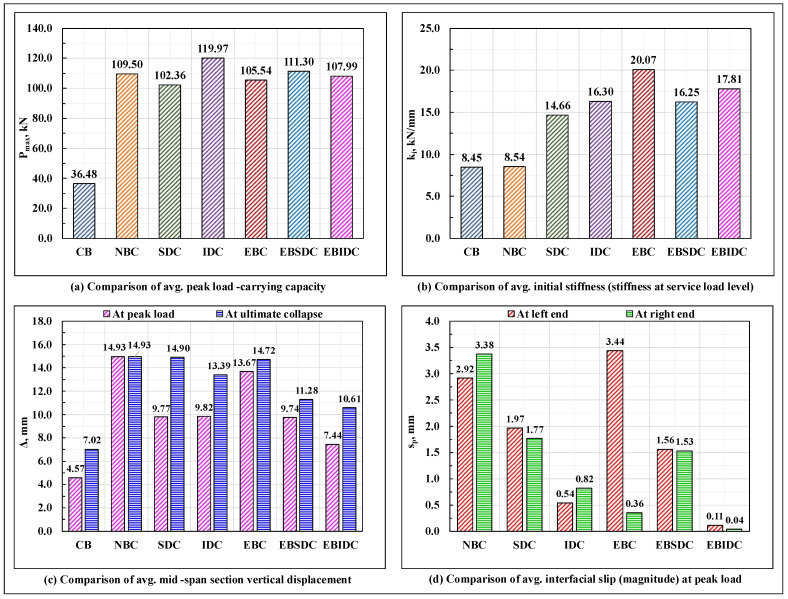
Comparison of the flexural response exhibited by the tested beam specimens.

The load-displacement response of the series #1 and #2 beam specimens is shown in Figure 11a,b, respectively; the vertical deflection profiles of the beam specimens at the peak load and at the moment of ultimate collapse, on average, are shown in Figure 11c,d, respectively; the bar charts displaying a relative comparison of the load-carrying capacity $\left(P_{max}\right)$, stiffness (initial stiffness) at the service load (${0.6P}_{max}$ load level), mid-span section vertical displacement at peak and ultimate collapse loads, and end slip (magnitude) at peak load, on average, of the tested beam specimens are plotted in Figure 12, as applicable. The critical parameters (peak load, initial stiffness, and end slip) of the tested series #1 and #2 beam specimens are shown in Table 4.

It can be observed that, amongst all the tested beam specimens, the EBC composite beams, as resulting from their relatively rigid interfacial connection, displayed the highest amount of initial stiffness, whereas the IDC composite beams, with their almost ideal display of composite action, possessed the maximum load-carrying capacity. The initial stiffness of the non-composite beams remained close to that of the standalone GFRP I-section beams, primarily due to the absence of the composite action (the two components spanned individually, and due to its relatively high deformability, the GFRP I-section component dominated the overall response); however, due to the differences in their failure mode(s), a significant difference in their corresponding load-carrying capacity was observed, where the CB specimens failed prematurely due to the local buckling of the compression, bearing failure/deformation/delamination of the top flange under the loading points, and crushing of the web occurring at the loaded sections; the provision of an unbonded concrete section over the compression flange of the GFRP I-section profile in the NBC specimens prevented the local failure of the compression flange and web of the GFRP I-section profile under the loading point and successfully mobilized its web to its longitudinal in-plane shear strength.

Relative to the NBC specimens, in the SDC and EBC composite beam specimens, the provision of a discrete shear connection at the GFRP-concrete interface (necessary for the structural integrity of the hybrid system and attainment of the composite action) almost doubled the initial stiffness of the composite beams but led to a marginal drop in their corresponding load-carrying capacities (primarily due to the increase in stiffness (which attracted relatively high stresses in the beam), excessive concentration of stresses in the concrete prism surrounding the dowels (which led to excessive cracking in the concrete section around the dowel) and at the bonded interface, weakening of the compression flange of the GFRP I-section profile due to the drilling of holes, a sudden spike in the horizontal shear stresses in the portion of the top web-flange junction of the GFRP I-section profile lying between the two dowel containing sections, as applicable). On the other hand, providing an inclination of 45° to the otherwise straight dowel provided a better anchorage to the dowels and reduced the concentration of bearing stresses in the concrete wedge surrounding the dowel; this prevented the concrete slab from undergoing excessive premature cracking and inhibited a sudden drop in stiffness, as observed previously in the SDC specimens, altogether enhancing their load-carrying capacity and initial stiffness relative to the non-composite beams.

Compared to their straight dowel counterparts (SDC composite beams), the IDC composite beam specimens provided with 45° inclined GFRP studs displayed an ideal composite action till failure (leading to a better utilization of the constituent materials), experienced less amount of interfacial slip at the beam ends (Figure 12d), had a comparable initial stiffness, and possessed a marginally high load-carrying capacity. However, when provided with an additional adhesive layer at the GFRP-concrete interface (over a wet concrete surface), in contradiction to their epoxy bond and straight dowel connection-based composite beam counterparts (EBSDC composite beams), where relative to their standalone counterparts (SDC composite beams), the presence of a secondary adhesive layer at the GFRP-concrete interface marginally enhanced both the load-carrying capacity and the initial stiffness (though the overall response in terms of the composite action, failure modes, and end slip remained almost invariant); in specimens with 45° inclined studs, adding a secondary adhesive layer at the GFRP-concrete interface reduced the interfacial slip to nearly zero and enhanced the initial stiffness, but considerably reduced the load-carrying capacity of the beam.

As previously explained in Section 3.1.6 and Section 3.1.7, except for the marginal contribution in enhancing the initial stiffness and reducing the interfacial slip, as apparent from the visual inspection, applying an epoxy-based adhesive layer over a wet concrete surface at the GFRP-concrete interface appears primarily ineffective, as it gets infused with the concrete matrix, causes honey-combing of the concrete layer at the GFRP-concrete interface upon compaction, and does not effectively bond with the surface of the GFRP I-section profile laminate (confirmations regarding this require further investigations at the microstructural level, such as scanning electron microscope (SEM) images, Digital Image Correlation (DIC) techniques, etc.). Fabricating composite beams with a joint connection of GFRP dowels and an adhesive layer applied to a hardened concrete surface appears practically challenging, primarily in terms of embedding the dowels in the concrete slab and connecting this prefabricated system over the compression flange.

**Figure 13:**
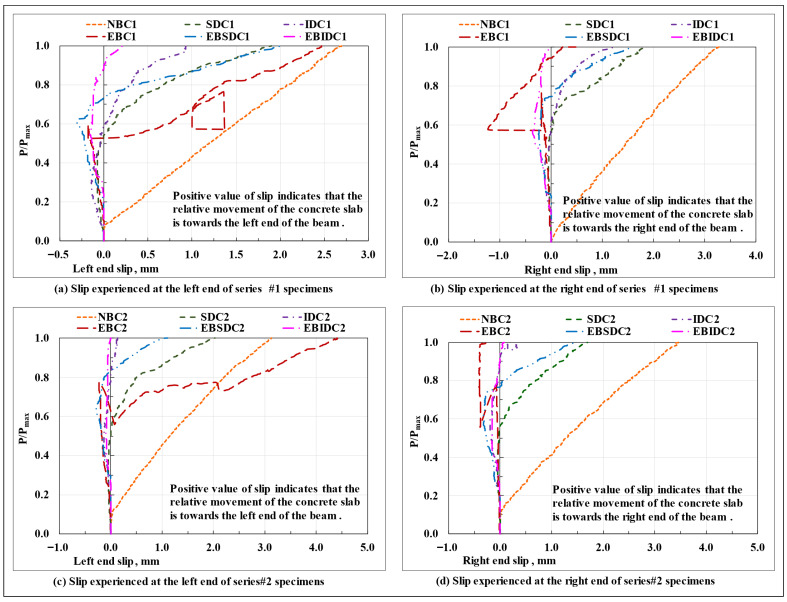
Load ratio–end slip plots of the tested composite and non-composite beam specimens.

Except for the epoxy-bonded connection-based composite beam specimens (EBC), in all other tested specimens, an almost equal amount of interfacial slip was experienced at two ends of the beam, thereby representing an almost uniform movement of the concrete slab relative to the connected flange of the GFRP I-section profile in both directions along the curvature of the bent beam, as possibly caused due to the formation of an identical state of interfacial stresses and subsequent undifferentiated deformation of the interfacial connectors in the two halves of the beam span. On the contrary, in the EBC composite beam specimens, as resulting from the non-uniform/uneven/progressive debonding/peeling/shearing of the adhesive layer and/or cohesive failure of the concrete surface close to the bonded interface with the increase in load, the concrete slab appears to have got partially anchored from moving in one of the span directions and advanced towards the other (which also explains the significantly high difference observed between the slip values recorded at the two ends of the EBC composite beam (Figure 12d)). The load ratio-end slip plots of the series #1 and series #2 composite beam specimens (Figure 13) show that the slip experienced by the tested EBC composite beam specimens was almost close to zero till the point of initiation of failure in the bond layer; however, resulting from the progressive loss of composite action (complete debonding of the concrete section) at varied sections along the beam span and the sudden separation of the concrete slab from the bonded flange of the GFRP I-section profile at the point of ultimate failure (total debonding or a complete loss of composite action at all sections of the beam), the total end slip (at least at one of the beam ends) exceeded that of the non-composite beams and all other composite beams examined in the present study, where, resulting from either a complete absence of any hindering medium at the interface or in case of the presence of the same the uniform/identical deformation of the shear connectors in the two halves of the beam, a gradual, symmetric, and uniform movement of the concrete slab (relative to the connected flanged of the GFRP I-section profile) occurred along the longitudinal axis of the beam throughout the test.

In the EBC composite beams, which were comparatively the stiffest among the others, the microstructure level damage at the bottom web-flange junction in the pure bending moment region was found to occur at the normal strain value that was lowest among all the tested beams. On the other hand, in the NBC specimens, which, in comparison to all the other composite beam specimens, possessed the least amount of initial stiffness, this critical normal strain value was the highest. Also, in contrast to the composite beams with a standalone interfacial connection of shear studs, in those provided with an additional adhesive layer (that led to a marginal rise in the beam stiffness), a sharp decline in the magnitude of this normal strain value was recorded; however, a direct correlation of this phenomenon with the initial stiffness of the composite beam could not be firmly established with the findings of the present study, and other factors, like the composite lay-up and the ply stacking sequence of the GFRP I-section profile laminate, magnitude/nature of debonding/delamination/peeling forces at the mid-span section bottom web-flange junction, modes of progressive failure of the laminate, etc. may influence the occurrence of the same. Confirmations regarding this require further investigations at the microstructural level, such as scanning electron microscope (SEM) images, Digital Image Correlation (DIC) techniques, etc.

## 4. Analytical Study

For composite beams that experienced failure primarily in the form of the longitudinal in-plane shearing of the GFRP profile web, analytical equations (Equations (1)–(9) [56]) developed by Zou et al. [56] were used for correlating the experimentally obtained test results (peak load, $P_{max}$). The equations were derived on the basis of the plane section assumption (i.e., the effect of shear deformations on the overall response of the composite beam was ignored) and the transformed section approach for the composite beams with a joint connection of stay-in-place formwork (as a shear key) and steel bolts, and a span length-to-overall depth ratio of ~9. The stiffness of the connector (derived from the corresponding push-out test) was taken as the critical parameter. The equations were validated and calibrated against the results of previously conducted and reported experimental studies on FRP-concrete composite beams with a wide variety of interfacial shear connections, such as steel bolts, epoxy resin layers, shear keys, etc., experiencing a similar failure mode.

(1) $$P_{max}=\frac{2}{\eta_{SD}\eta_{f}}A_{web}\tau_{12}$$

(2) $$\eta_{SD}=\frac{\tau_{max}}{\tau_{avg}}$$

(3) $$\eta_{F}=\frac{1-m_{o}}{1+\frac{1}{12\alpha_{E}\alpha_{1}\alpha_{2}^{2}}}+\frac{m_{o}}{1+\alpha_{2}}$$

(4) $$m_{o}=\left\{\begin{array}{c} \frac{h_{o}^{2}}{A_{1}}\left(1-sech\left(\frac{\alpha L}{2}\right)cosh\left[\alpha \left(\frac{L}{2}-b\right)\right]\right)\;:\;Partial\;composite\;action\\ \frac{h_{o}^{2}}{A_{1}}\;\;\;\;\;\;\;\;\;\;\;\;\;\;\;\;\;\;\;\;\;\;\;\;\;\;\;\;\;\;\;\;\;\;\;\;\;\;\;\;\;\;\;\;\;\;\;\;\;\;\;\;\;\;\;\;\;\;\;\;\;\;\;\;:\;Full\;composite\;action\;\;\;\;\;\; \end{array}\right.$$

(5) $$A_{1}=\frac{I_{o}}{A_{o}}+h_{o}^{2}$$

(6) $$A_{o}=\frac{A_{F}A_{c}}{\alpha_{E}A_{F}+A_{c}}$$

(7) $$I_{o}=\frac{I_{c}}{\alpha_{E}}+I_{F}$$

(8) $$\alpha =\sqrt{\frac{kA_{1}}{E_{F}I_{o}}}$$

(9) $$k=\frac{rK_{s}}{q_{s}}$$

where, $A_{web}$ represents the cross-sectional area of the GFRP I-section web; $\tau_{12}$ is the longitudinal in-plane shear strength of the GFRP I-section web; $\eta_{SD}$ is the ratio of the maximum $\left(\tau_{max}\right)$ and the average $\left(\tau_{avg}\right)$ shear stresses developed in the GFRP I-section web at failure (corresponding to the peak load $\left(P_{max}\right)$; from a detailed parametric study and validation of previously conducted experimental results, as communicated in the various literature sources, an average value of 1.41 was assigned to $\eta_{SD}$ by the authors [56]); $\eta_{F}$ is a dimensionless factor that represents the contribution of the web area relative to that of the concrete slab in resisting the shear stresses; $m_{o}$ is a dimensionless factor that considers the type of loading configuration, the type of composite action (full/partial), and the connection stiffness; $\alpha_{E}$ represents the modular ratio $\left(E_{F}/E_{c}\right)$; $E_{F}$ and $E_{c}$ are the longitudinal elastic modulus of the GFRP I-section profile (taken as equal to the longitudinal elastic flexural modulus $\left(E_{1f}\right)$ of the GFRP I-section profile) and the concrete in the slab, respectively; $\alpha_{1}$ and $\alpha_{2}$ represent the ratio of the joint cross-section area of both GFRP I-section flanges to that of the concrete slab and the ratio of the overall depth of the GFRP I-section to that of the concrete slab, respectively; $h_{o}$ is the average (arithmetic mean) of the thickness of the concrete slab and the overall depth of the GFRP I-section; L is the effective span; b is the length of the shear span; $A_{F}$ and $A_{c}$ are the cross-sectional area of the GFRP I-section and the concrete slab, respectively; $I_{F}$ and $I_{c}$ are the moment of inertia of the GFRP I-section and the concrete slab about their respective centroidal axis, respectively; $K_{s}$ is the stiffness of a single connector; r is the no. of connectors placed in the transverse direction at a particular section; and $q_{s}$ is the longitudinal c/c spacing of the connectors.

Based on the value of the connector’s stiffness (obtained from the corresponding push-out tests), the above-mentioned equations were deployed to calculate the theoretical $P_{max}$ value for the SDC, IDC, and EBIDC (almost full composite action till the peak load) beam specimens. Calculations for the EBC and EBSDC were not carried out because, in the EBC specimens, a full composite action was not maintained till the peak load (as was assumed in the analytical equations for a continuously bonded system), and failure was observed in the form of the crushing of the web at the pure bending moment region; the behavior of EBSDC was found to be similar to that of the SDC specimen (also, the corresponding push-out test results were not available). The results of the analytical study and the values of the corresponding key parameters are summarized in Table 5.

It can be observed that, since the available equations (derived on the basis of the Euler-Bernoulli beam theory) ignored the effect of shear deformations in the GFRP section and were derived and validated/calibrated primarily with/against the results of experimental studies concerning a relatively ductile connection of steel studs, shear keys, stay-in-place formwork, etc., a value of 1.41 for the factor $\eta_{SD}$ appears inadequate for the present case of composite beams with a low L/D ratio (~5) and relatively brittle epoxy resin-saturated glass roving plugs based on GFRP stud connectors. The equations underestimated the load-carrying capacity of the SDC composite beams by around 23% and that of the IDC composite beams by about 36%; on the other hand, in the case of composite beams with an almost full/rigid connection till the peak load (EBIDC specimens), it was overestimated by around 16%. Based on the results of the conducted experimental study, for each of the considered cases of composite beams and shear connectors, the parameter $\eta_{SD}$ was re-evaluated (back-calculated) (last column of Table 5). However, confirmations regarding the generalization of the same and the derivation of analytical expressions taking into consideration the high shear deformation effects, formulation of a simplified design approach, and proposal of design aids, by taking into consideration all possible failure modes and their propagation mechanisms, the effects of different geometric, configurational, and material parameters, and a detailed analysis/discussion of the same, need further investigations.

## 5. Conclusions

The paper discusses the findings of an experimental study conducted to examine the flexural response of epoxy resin-saturated glass roving tied GFRP dowel connector-based composite beams with low L/D ratios. The key takeaways from the conducted research are as follows:

1. The proposed configurations of composite beams successfully prevented the premature failure in the GFRP I-section structural profile and, on average, tripled its load-carrying capacity and doubled the initial stiffness.
2. In contrast to the non-composite beams, the composite beams experienced a certain amount of lag between the shear failure of the web and its subsequent buckling/crushing under the loading points; this led to the formation of a secondary rising arm in the load-displacement curve and imparted a certain level of reserved capacity and ductility to the otherwise brittle system.
3. The composite beams experienced failure in the form of cracking/crushing of the concrete slab, progressive delamination of the GFRP laminate, debonding of the adhesive layer, shearing of the web at/near the top web-flange junction, and crushing/buckling of the web under the loading points.
4. No signs of damage/repositioning of the studs were observed in any of the tested mechanically connected composite beams. The proposed innovative method of riveting down the dowels using repeated wounds of epoxy resin-saturated glass roving proved efficient in transferring the interfacial shear stresses and resisting the uplifting forces till the ultimate failure of the beam.
5. Among all the developed beam configurations, the epoxy-bonded composite beams displayed the highest amount of initial stiffness, while those with a 45° inclined configuration of dowels, resulting from the attainment of an ideal composite action till failure, exhibited the highest load-carrying capacity.
6. Deploying an epoxy-based resin layer over the wet concrete surface, together with GFRP studs, appeared ineffective, as the wet epoxy got infused in the concrete matrix, caused honey-combing of the concrete layer at the GFRP-concrete interface upon compaction, and did not effectively bond with the GFRP laminate.
7. In none of the tested composite beams at failure the highly stressed extreme fibers of the maximum bending moment section attained the respective failure strain value. Also, in the same region, microstructural-level damage was observed in the bottom web-flange junction at low load levels. Hence, for an effective utilization of the material strength, the web-flange junctions need to be strengthened along the entire span length.
8. The Euler-Bernoulli beam theory-based analytical equations for predicting the peak load of FRP-concrete hybrid beams failing in shear, need to be accordingly modified for the case of high shear deformable composite beams provided with a brittle interfacial connection.

## Figures and Tables

**Table 1:** Specimen nomenclature code details.

Specimen Id	Test Series	Specimen Details
CB1	1	Control beam
CB2	2	
NBC1	1	Non-composite beam
NBC2	2	
SDC1	1	Composite beam with straight dowel connectors
SDC2	2	
IDC1	1	Composite beam with 45° inclined dowel connectors
IDC2	2	
EBC1	1	Composite beam with epoxy bond connection
EBC2	2	
EBSDC1	1	Composite beam with a combined connection of epoxy bond layer and straight dowel connectors
EBSDC2	2	
EBIDC1	1	Composite beam with a combined connection of epoxy bond layer and 45° inclined dowel connectors
EBIDC2	2	

**Table 3:** Summary of the four-point bending test results.

Specimen	Peak Load	Mid-Span Section Vertical Displacement at Peak Load	Mid-Span Section Vertical Displacement Capacity *	Initial Stiffness ^#^	Interfacial Slip (Magnitude) at Peak Load		Primary, Ultimate Mode(s) of Failure ^$^
	$P_{max}$ (kN)	$\Delta_{p}$ (mm)	$\Delta_{u}$ (mm)	$k_{i}$ (kN/mm)	At the Left Beam End, $s_{p,left}$ (mm)	At the Right Beam End, $s_{p,right}$ (mm)	
**CB**	36.48	4.57	7.02	8.45	-	-	Local buckling of the compression flange, bearing failure/deformation of the top flange, and crushing of the web under the loading points
**NBC**	109.50	14.93	14.93	8.54	2.92	3.38	Longitudinal in-plane shearing of the top web-flange junction and simultaneous buckling of the web under one of the loading points
**SDC**	102.36	9.77	14.90	14.66	1.97	1.77	Longitudinal in-plane shearing of the top web-flange junction, followed by a delayed buckling of the web under one of the loading points
**IDC**	119.97	9.82	13.39	16.30	0.54	0.82	Longitudinal in-plane shearing of either top or both top and bottom web-flange junctions, followed by either a simultaneous or delayed buckling of the web under one of the loading points
**EBC**	105.54	13.67	14.72	20.07	3.44	0.36	Crushing of the upper half of the web, followed by a complete separation of the bonded components
**EBSDC**	111.30	9.74	11.28	16.25	1.56	1.53	Longitudinal in-plane shearing of the web near the top web-flange junction, followed by either a simultaneous crushing of the upper half of the web or a delayed buckling of the web under one of the loading points
**EBIDC**	107.99	7.44	10.61	17.81	0.11	0.04	Longitudinal in-plane shearing of the top web-flange junction, followed by a delayed buckling of the web under one of the loading points

* Corresponding to the load level at/close to the ultimate/final collapse of the beam. ^#^ Measured at service load level (for the tested composite beams, the secant stiffness at ${0.6P}_{max}$ load level roughly corresponded to the typical service load mid-span section vertical deflection limit of L/250 [54]). ^$^ Primary mode(s) of failure correspond to the failure mode(s) experienced at/close to the peak load level $\left(P_{max}\right)$; ultimate mode(s) of failure correspond to the failure mode(s) experienced at/close to the ultimate/final collapse of the beam.

**Table 4:** Critical parameters of the tested series #1 and #2 beam specimens.

Parameter	Test Series	CB	NBC	SDC	IDC	EBC	EBSDC	EBIDC
**Peak load,** $P_{max}$ **(kN)**	*#1*	34.92	110.51	101.57	126.22	103.36	115.39	106.12
	*#2*	38.05	108.50	103.15	113.72	107.72	107.20	109.87
	$\mathrm{Mean},\;\bar{x}$	***36.48***	***109.50***	***102.36***	***119.97***	***105.54***	***111.30***	***107.99***
	$\mathrm{Standard}\mathrm{deviation},\;s_{n-1}$	*2.21*	*1.42*	*1.12*	*8.84*	*3.08*	*5.79*	*2.65*
**Initial stiffness (at service load level),** $k_{i}$ **(kN/mm)**	*#1*	8.55	8.47	15.08	15.69	19.80	16.07	17.45
	*#2*	8.36	8.61	14.24	16.92	20.34	16.43	18.16
	$\mathrm{Mean},\;\bar{x}$	***8.45***	***8.54***	***14.66***	***16.30***	***20.07***	***16.25***	***17.81***
	$\mathrm{Standard}\mathrm{deviation},\;s_{n-1}$	*0.13*	*0.10*	*0.60*	*0.86*	*0.38*	*0.26*	*0.50*
**Interfacial slip (magnitude) at the left end of the beam at peak load, ** $s_{p,left}$ **(mm)**	*#1*	-	2.71	1.90	0.95	2.47	2.01	0.22
	*#2*	-	3.13	2.03	0.13	4.40	1.11	0.00
	$\mathrm{Mean},\;\bar{x}$	**-**	***2.92***	***1.97***	***0.54***	***3.44***	***1.56***	***0.11***
	$\mathrm{Standard}\mathrm{deviation},\;s_{n-1}$	-	*0.30*	*0.09*	*0.58*	*1.36*	*0.64*	*0.16*
**Interfacial slip (magnitude) at the right end of the beam at peak load, ** $s_{p,right}$ **(mm)**	*#1*	-	3.28	1.83	1.29	0.48	1.58	0.00
	*#2*	-	3.48	1.70	0.34	0.23	1.48	0.08
	$\mathrm{Mean},\;\bar{x}$	**-**	***3.38***	***1.77***	***0.82***	***0.36***	***1.53***	***0.04***
	$\mathrm{Standard}\mathrm{deviation},\;s_{n-1}$	-	*0.14*	*0.09*	*0.67*	*0.18*	*0.07*	*0.06*

**Table 5:** Comparison of experimental and theoretical results; recommended recalculated values of $\eta_{SD}$.

Specimen Id	$K_{s}$, (kN/mm)	$m_{o}$	$\eta_{F}$	$P_{max}$, (kN)		Pmax,theoryPmax,exp.	$\eta_{SD}$ (Recalculated)
				Theory *	Experiment		
SDC	2.27	0.183	0.773	79.10	102.36	0.77	1.089
IDC	1.79	0.153	0.791	77.30	119.97	0.64	1.000
EBIDC (full composite action)	-	0.645	0.487	125.56	107.99	1.16	1.639

* Based on the suggested $\eta_{SD}$ value of 1.41 [56].

## Data Availability

The raw data supporting the conclusions of this article will be made available by the authors on request.

## Abbreviations

The following abbreviations are used in this manuscript:

FRP	Fiber-reinforced polymer
GFRP	Glass fiber-reinforced polymer
CFRP	Carbon fiber-reinforced polymer
CB	Control beam
NBC	Non-composite beam
SDC	Straight dowel connection based composite beam
IDC	45° inclined dowel connection based composite beam
EBC	Epoxy-bonded composite beam
EBSDC	Epoxy bond and straight dowel connection based composite beam
EBIDC	Epoxy bond and 45° inclined dowel connection based composite beam
